# Pathways to Enable Primary Healthcare Nurses in Providing Comprehensive Primary Healthcare to Rural, Tribal Communities in Rajasthan, India

**DOI:** 10.3389/fpubh.2020.583821

**Published:** 2020-11-27

**Authors:** Arpita Amin, Manisha Dutta, Sanjana Brahmawar Mohan, Pavitra Mohan

**Affiliations:** ^1^Basic Healthcare Services, Udaipur, India; ^2^Primary Healthcare Initiative (Joint Partnership of Indian Institute of Management Udaipur and Basic Healthcare Services), Udaipur, India

**Keywords:** primary care, community health, rural pathway, task redistribution, primary healthcare nurses

## Abstract

**Background:** Emerging health needs and uneven distribution of human resources of health have led to poor access to quality healthcare in rural areas. Rural pathways provide an approach to plan and evaluate strategies for ensuring availability, retention, motivation, and performance of human resources for health in rural areas. While effectiveness of primary healthcare (PHC) nurses to deliver primary health care is established, there is not enough evidence on ways to ensure their availability, retention, motivation, and performance. The paper draws on the program experience and evidence from a primary healthcare network (AMRIT Clinics), in which nurses play a central role in delivering primary healthcare in rural tribal areas of Rajasthan, India, to bridge this gap.

**Methods:** Rural, tribal areas of Rajasthan have limited access to functional healthcare facilities, despite having a high burden of diseases. We used the rural pathway approach to describe factors that contributed to the performance of the nurses in AMRIT Clinics. We analyzed information from the human resource information system and health management information system; and supplemented it with semi-structured interviews with nurses, conducted by an independent organization.

**Results:** Most nurses were sourced from rural and tribal communities that the clinics serve; nurses from these communities were likely to have a higher retention than those from urban areas. Sourcing from rural and tribal communities, on-going training in clinical and social skills, a non-hierarchical work environment, and individualized mentoring appear to be responsible for high motivation of the primary healthcare nurses in AMRIT Clinics. Task redistribution with due credentialing, intensive and on-going training, and access to tele-consultation helped in sustaining high performance. However, family expectations to perform gendered roles and pull of government jobs affect their retention.

**Conclusion:** Rural and remote areas with healthcare needs and scarcity of health provisions need to optimize the health workforce by adopting a multi-pronged pathway in its design and implementation. At the same time, there is a need to focus on structural factors that affect retention of workforce within the pathway. Our experience highlights a pathway of up-skilling PHC nurses in providing comprehensive primary healthcare in rural and remote communities in Low and Middle-Income Countries (LMICs).

## 1. Introduction

India has a population of 1.3 billion, about 2/3rd of which resides in rural areas (1), about 1 in 10 belong to one of the many scheduled tribes (2). Rural and tribal communities face a higher disease burden than others, but have much less access to healthcare (2). Poor access to healthcare for these communities is often on account of uneven distribution of qualified health providers between rural and urban areas; and between tribal and non-tribal areas. According to *The Health Workforce in India* report (2016), there are almost four times as many doctors and nurses in urban areas than rural areas (3). Moreover, high levels of absenteeism (4), low motivation marked by social and family isolation, poor educational opportunities for children, insufficient pay (5) affect quality of healthcare in these areas.

Task-shifting is one of the strategies used to enhance availability of skilled human resources in rural areas. The World Health Organization (WHO) defines task-shifting as: “*Specific tasks are moved, where appropriate, from highly qualified health workers to health workers with shorter training and fewer qualifications in order to make more efficient use of the available human resources for health*” (6). An emerging task-shifting strategy is up-skilling of nurses to undertake larger responsibilities than their historically defined role of assisting a physician. Task-redistribution is used synonymously to task-shifting, and we have preferentially used the former nomenclature.

Previous reviews highlight the effectiveness of up-skilling nurses in meeting healthcare demands amidst growing burden of diseases (7–10). The reviews clearly establish that when skilled, mandated, and supported, nurses are as effective as doctors in delivering primary health care. Higher patient satisfaction outcomes (7, 8) have been observed with nurses as first point of contact (11) and thus patients more likely to keep their follow-up appointments with the nurses (12). This satisfaction is also associated with greater engagement with the patient (13) through counseling, two-way communication and drawing in context-specific real life connect (14). Nurse-led clinics (15) have been effective in providing specialized curative care and in improving patient outcomes.

Rural Pathways refer to a multi-pronged approach taken to sustain efforts to improve health outcomes in rural areas. Rural pathways may be designed to address specific needs identified in the community like the scarcity of health workforce in rural areas. O'Sullivan et al. (16) drew evidence and came up with a comprehensive checklist for implementing rural pathways for rural workforce in low- and middle-income countries (LMICs). The checklist highlights several components that initiate from the community needs to health worker selection, training and up-skilling, accreditation, and recognition as well as monitoring of outcomes. Examples from LMICs which were key to the lessons drawn for the rural pathway checklist focussed largely on availability of physicians or on community volunteers' up-skilling to perform certain primary care functions in rural areas.

A significant proportion of task-redistribution and nurse-led healthcare interventions have been implemented and tested in high-income countries. Also, most research focuses on assessing effectiveness outcomes of nurse-led interventions. However, there is limited understanding of what it takes to ensure availability, retention and ensuring high performance of nurses in delivering primary health care, especially in rural and tribal areas. Several systemic barriers such as hierarchies in the workforce and structural barriers and gender inequality pose as barriers in enabling nurses (most of whom are women), to assume leadership roles in countries like India (17, 18).

There is a critical need for exploring rural pathways that ensure availability, retention, and effectiveness of nurses as primary healthcare providers in rural areas in LMICs. Basic Healthcare Services (BHS), a not-for-profit organization that authors are associated with, runs a network of six primary healthcare clinics (called AMRIT Clinics) in rural and tribal areas of South Rajasthan, India. The clinics are managed by Primary healthcare (PHC) nurses, supported by a visiting and on-call primary care physician at one end, and community health workers and volunteers on the other. We analyzed our evidence and experience of running these Clinics to identify what steps along the rural pathways are required to ensure availability and effectiveness of PHC nurses in delivering primary healthcare in such areas.

## 2. Methodology

### 2.1. Context

Southern region of Rajasthan is predominantly rural, most of which is inhabited by a population belonging to one or other scheduled tribes (19). The region has the lowest Human Development Index (HDI) of 0.5 (20). Only about 35% of women in these communities are literate (1). From as many as 45% households, at least one man migrates to cities in search of livelihood (21). Half of all children are underweight and a similar proportion of mothers are malnourished too (20). Absence of functional health facilities in the area due to high rates of absenteeism (4), poor availability of food (19), and distress migration have a detrimental impact on the health status of the population in these areas, especially women and children. Careseeking (22) among these households remain erratic with only 6% of households seeking healthcare from formal qualified providers; others resorting to informal providers or traditional healers (4%).

### 2.2. Pathway Framework

**Figure 1:**
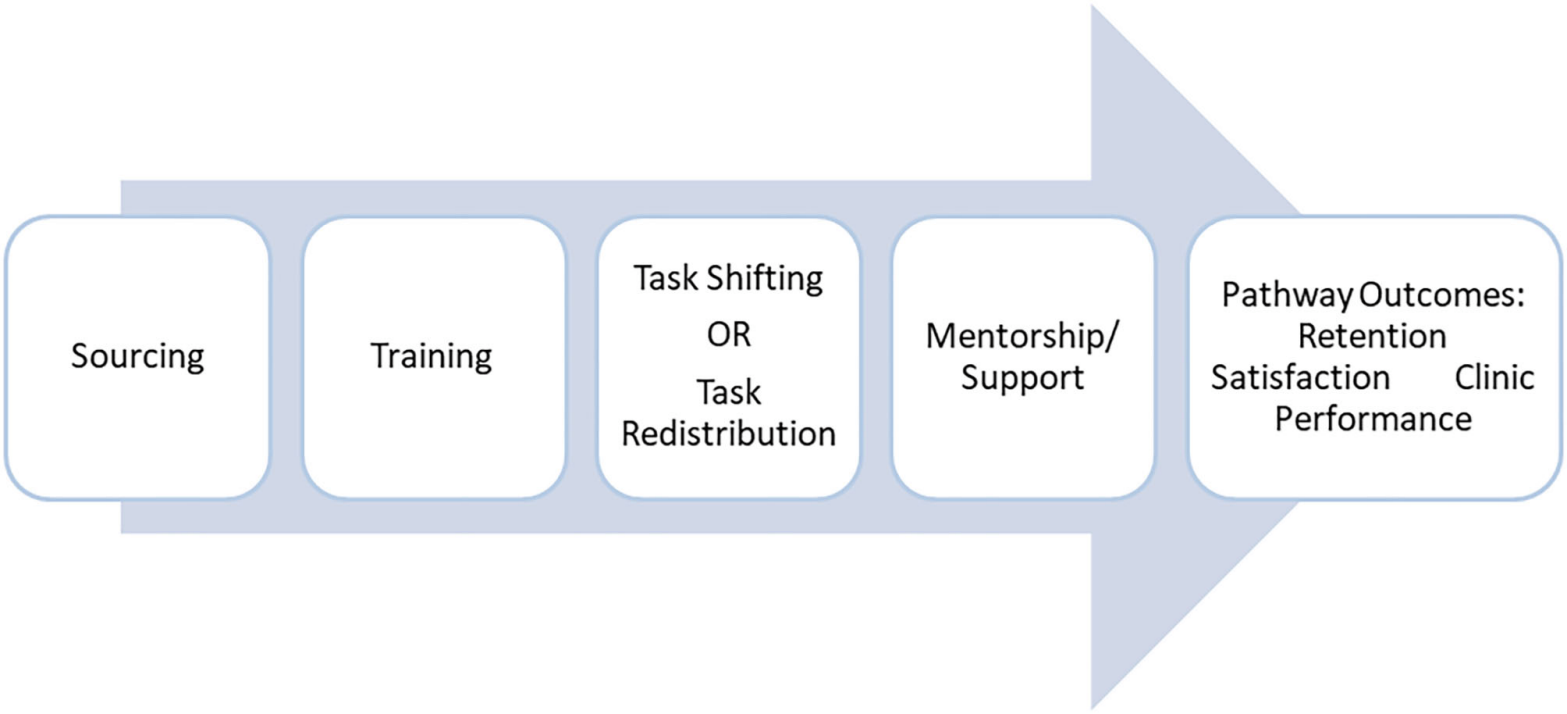
Pathway to enable nurses to provide primary healthcare in rural areas.

To evaluate the effectiveness of strengthening capacities of PHC nurses, we adapted the existing rural pathway framework (16) and contextualized it (Figure 1). The components of the Pathway are described in Table 1.

**Table 1:** Description of the steps in the Pathway.

**Description of the pathway components**	
Sourcing	Sourcing refers to the selection and recruitment of nurses done based on demographics, qualification, and experience
Training	Trainings are intended to keep the nurses' knowledge and practice updated.
Task shifting or task redistribution	Task shifting is the “*rational redistribution of tasks among health workforce teams*” (6)
Mentorship/support	Mentorship and support include ways in which nurses are supported to perform and grow personally and professionally
Outcome	(i) Retention: Duration for which nurses continue to be in their current jobs
	(ii) Satisfaction: A positive perception of the nurses on their role
	(iii) Performance: Effectiveness in meeting health needs of the populations served

### 2.3. Methods for the Study

To assess the effectiveness of the pathway and to evaluate the development outcomes, secondary data from three sources was analyzed. The sources of data for each pathway component are explained in Table 2.

**Table 2:** The sources of data for the Pathway Components.

**Sources of data for the pathway components**		
Sourcing	Organization's Human Resource Information System	Nurses' qualification, background and place of origin data.
Training		The number of trainings conducted and the subject of trainings
Data on retention and attrition		The duration spent by each nurse at the clinic and the reasons for leaving.
Data on nurse's satisfaction	Qualitative data was accessed from nurses' interviews conducted by a third party organization named “Start Up!” for need assessment of leadership training (interviews conducted in February 2020)	Quotes suggestive of the themes identified in the interviews.
Data on patients managed	Organization's Health Management Information System (HMIS) (data retrieved for the years 2015–2019)	The data on the different types of cases managed by the nurses and the doctors.

The quantitative data was analyzed in MS Excel, using pivot tables to segregate provider level data and health condition wise data. For qualitative data, interview verbatim from a previous source was accessed, translated, and analyzed while keeping the names of the respondents anonymous. Emerging themes were identified, and supporting quotes were elicited to substantiate the themes.

## 3. Results

We analyzed information from above sources to describe and assess the different components of rural pathways for PHC nurses.

### 3.1. Sourcing

Nurses registered with Indian Nursing Council; with either a Bachelor of Science in Nursing (BSc Nursing) or a Diploma in General or Auxiliary Nursing and Midwifery (GNM or /ANM) were eligible for recruitment as PHC nurses in AMRIT Clinics. An affirmative action was taken to source them from rural and tribal areas, as they are more likely to live and stay in such areas and more empathetic to needs of the communities.

A total of 40 PHC nurses have been recruited in AMRIT Clinics from September 2012 till May 2020. Of these, 93% belonged to rural areas and 83% of them come from similar tribal communities that they serve (Table 3). Eighty-two percent of them had a diploma in GNM and 12.5% had a diploma in ANM. Two (5%) were interns pursuing a GNM degree.

**Table 3:** Place of origin of Nurses.

	**Non-tribal**	**Tribal**	**Total**
Rural	5(13.5)	32 (86.5)	37 (92.5)
Urban	2 (66. 7)	1 (33.3)	3 (7.5)
Total	7 (17.5)	33 (82.5)	40

### 3.2. Training

All nurses recruited as PHC Nurses undergo a focused one-month induction training, followed by 3–6 months intensive on-the-job training at clinics. This is followed by continuous nursing education, 2–3 days per month.

The training themes span across clinical knowledge and skills, communication and other soft skills, and management skills. Each component also emphasizes on context-specific understanding of social and cultural determinants of health. In addition, weekly primary care physician's visits and bi-monthly visits by Nurse Mentors are also opportunities to train on new and refresh on older learnings.

“*There is a good balance between training on clinical issues and (exposure to) social realities we see*” shares a Nurse Coordinator about the training imparted to them.

### 3.3. Task Redistribution

Task redistribution is a key component of the pathway that enables PHC Nurses to perform their duties effectively. In AMRIT Clinics, PHC Nurses are entrusted and credentialed to perform the primary curative functions, and enabled to do so through continuous training, standardized protocols, point-of-care diagnostics, and easy access to tele-consultation. PHC nurses reside in the Clinic villages and provide care on all days. Primary care physician lives in the nearby town, visits the clinics weekly on designated days, and is available for tele-consultation at all times. Redistributing the tasks of primary curative care between PHC nurses and the physician does not equate to shifting of responsibility: the responsibility is jointly shared by the two.

“*When a patient comes with a condition we are not certain, we call the consult the doctor on telephone. We also ask the patient to come on the designated day for doctor's visit after assessment. This is how wee ensure that no patient returns without receiving any counsel*” says a Nurse about the patient management procedure.

### 3.4. Support and Mentoring

The organization (BHS) supports PHC Nurses to play their role and mentors them for their personal and professional growth. Physicians provide mentoring and tele-consultation support to the PHC Nurses. Regular monitoring of data and feedback is provided to the clinic teams for quality of care.

Career advancement opportunities are provided to the PHC nurses: six of them have so far advanced to Nurse Coordinator position, and one of them has progressed to the Nurse Mentor position. The Nurse Mentor and a Clinic Associate supervise and mentor the PHC nurses individually through periodic on-site visits and through on-line support. The mentors identify opportunities for their personal and professional growth and assist them to tap those opportunities.

Additionally, an accommodation with basic amenities such as water, electricity, and sanitation; a competitive salary (comparable to salary in city hospitals) and a well-equipped clinic with an amicable and respectful environment is made available to all of them.

### 3.5. Pathway Outcomes

#### 3.5.1. Work Satisfaction and Motivation

Although no formal investigation was conducted to assess the job satisfaction, an independent organization conducted qualitative interviews with six PHC nurses to assess their leadership potential and needs for training. Interviews revealed a high sense of pride and satisfaction they perceived in managing the patients independently, equivalent to that of a physician:

“*We used to work under the doctor in the clinic but here we ourselves, it feels nice that we are doing doctor's work also*,” shares a PHC nurse at one of the clinics.

Nurses appear to have a high level of patient-centeredness, as illustrated by the following quote by a nurse who was interviewed:

“*We do not work for money here; days merge in nights, but we try to ensure that the patients get better*.”

High motivation appears to be also on account of the team spirit: “*Our team of doctors and nurses have always been supportive which keeps me motivated*.”

“*We feel gratified when we see the change in the community, it makes us forget all the fatigue from the hardwork*,” shares a nurse about her work in AMRIT Clinics.

#### 3.5.2. Retention

A total of 38 PHC nurses have been recruited in AMRIT over the last 7 years. Of those, 20 left the job after spending a median of 15 months (IQR: 8–35 months) A further analysis on segregating the PHC nurses based on their place of origin and caste found that the three from urban background areas stayed for a median duration of just over 6 months, while those from rural areas stayed for a median duration of 16 months.

Out of 38 PHC nurses employed so far, 20 left for various reasons. The two major reasons for leaving the job were: shifting to a government job (10), and for family reasons (10). The appeal of a “government” job is often what drives the PHC nurses' aspirations. As one of them shared:

“*If we get a government job then we can get a posting near our home. We are working with this thought that in government job the salary will be better and would help in supporting our family*.”

While the salary at AMRIT Clinics is comparable to the best of private hospitals in cities, salaries and perception of job-security for government jobs is much higher.

Table 4 shows the comparison between the remuneration and perks offered by the government, private hospitals, and AMRIT Clinics.

**Table 4:** Comparison of remuneration and perks offered by Government facilities, private hospitals and AMRIT Clinics.

**Indicator**	**Government Hospitals**	**AMRIT**	**Private Hospitals**
Salary	26500	12,400	12,000
Holidays	7 weekly holidays in a month	4 weekly holidays a month	4 weekly holidays a month
Other perks	Other benefits like provident fund and Insurance are identical across all institutions		

The average age of PHC nurses who have worked at the clinics was found to be 27 years, with the age range being 21–38 years. Of the 38 PHC nurses, 30 are married and more than 20 of them were mothers often with more than one child. The “family” reasons for leaving often pertained to child bearing or child rearing roles and presence at home for carrying out other household chores, such as care of ailing parents-in-law. As one of the PHC nurses puts it: “*We are staying away from our family. We cannot bring them here. I have not stayed with the family since my marriage*.” The reason for their families not staying with them is lack of employment opportunities for their spouses and a poor quality of schools in the villages.

#### 3.5.3. Performance

A retrospective analysis of the records from 2015 to 2019 of 3 AMRIT Clinics saw 87,227 patient visits, of whom 55,825 (64%) were women and 14,829 (17%) were children. While PHC nurses managed most of reproductive and child health conditions and communicable diseases, visiting physicians managed most of non-communicable diseases (Table 5).

**Table 5:** Clinic performance outcomes.

**Management of different health conditions at AMRIT Clinics**			
**Indicator**	**Total cases managed**	**Cases managed by PHC Nurses**	**Cases managed by Physicians**
All Illnesses	87,227	63,213 (62%)	24,014 (38%)
Reproductive health conditions	19,053	14,613 (77%)	4,440 (23%)
Childhood illnesses	10,452	7,253 (69%)	3,199 (31%)
Communicable diseases	25,964	15,059 (59%)	10,705 (41%)
Non-communicable diseases	9,287	3,835 (41%)	5,452 (59%)
Injuries	4,649	3,254 (70%)	1,395 (30%)

A major indicator of quality of the service provided at the clinics is trust amongst the community. Sixty-three percent of the total patients visits made were repeat visits at reflect their perception of the care they receive at the clinic. The PHC nurses are also equipped with a protocol to manage patients with an array of conditions and also Standard Operating Procedures (SOPs) to ensure that the operation of the clinics are uniform and of high quality across all clinics.

## 4. Discussion

Our analysis demonstrates that a rural workforce pathway approach can be effective in planning for and assessing the factors that contribute to availability, satisfaction, and performance of nurses to provide primary healthcare in rural underserved communities. While the components of such a pathway are similar to what have been described earlier for physicians (16), gender-based roles of female nurses and aspirations of government jobs emerged as additional factors to be mindful while planning and evaluating such pathways for PHC nurses.

The analysis showed that nurses from rural areas were likely to have a higher retention than those from urban areas. We also noted that these PHC nurses were more likely to have community connect, and performed better. Studies from other contexts have also demonstrated that healthcare staff are more likely to stay on in jobs if they belong to the similar communities they are expected to serve (23, 24); which in turn leads to their higher effectiveness (16).

At AMRIT Clinics, transition from an ANM or GNM to the role of primary healthcare nurse requires training to build the clinical acumen, understand the context and community needs and to build counseling and management skills. In preparing physicians to undertake rural health, there have been efforts to integrate in their training the values of empathy, social accountability, and community sensitivity (25). We have also observed the value of integrating these values within structured training as well as through mentoring and feedback provided to the PHC nurses.

While task-shifting and task redistribution have been used synonymously, we prefer the latter term, as it signifies the principles of equality (as opposed to hierarchy) and of shared responsibility and accountability. Task redistribution in AMRIT Clinics resulted in complementarity between roles of PHC nurses and physicians: while the former played a larger role in managing maternal and child health conditions and communicable diseases, physician played a role in managing non-communicable diseases, providing tele-consultation, as well as in training PHC nurses on weekly visits. Both contributed significantly to the clinic performance. Such an arrangement allows the PHC nurses to perform their role with confidence, and for the team to have high performance.

One of the key challenges in providing primary healthcare services in rural and remote areas is sustaining the services through retention and sustenance of healthcare providers. There is no uniform strategy that looks at what works and what does not. Literature on human resource for health (26, 27) highlights working conditions and job satisfaction as important for retention. Studies also emphasize on remuneration and career advancement as key strategies as well (8, 26). Our experience also elicits the value of these components in building self-confidence and motivation of the PHC nurses. Additionally, from our experience, it appears that an enabling work environment, simple-to-use technology, and on-going mentoring sustains the motivation of the PHC nurses.

The opportunity to undertake responsibilities over and above normative responsibilities within nursing practice is perceived as equivalent to a physician's work. Therefore, the pathway enables the aspirations and potentials of this cadre by also drawing in a valuable role of the physician in shaping it.

Studies on nurse practitioners in high-income countries have elucidated how they have been able to provide services equivalent to physicians and have been effective in providing specialized care such as NCDs, maternal health, etc. (8–10). In our context, we observed that when mandated, skilled, and supported, PHC nurses can meet the complex health needs of rural and tribal populations effectively. In fact, they are likely to promote equitable access to healthcare, by extending care to women and children.

Studies on human resource in health highlight several push and pull factors that are responsible for retention of the healthcare workforce in rural areas (26, 28). This ranges from working conditions, home and family factors, local environment, and the national and international environment. In our context, the three areas inhibit retention. Firstly, family pressure owing to gendered expectations of the PHC nurses to manage their homes, children, and therefore desired proximity of the workplace closer to their home. Secondly, poor infrastructure of opportunities in the local environment such as housing for spouses, education facilities for children. Thirdly, in the context of India, a pull factor is the desire for a government job due to a higher remuneration offered for permanent nurses and job security.

Based on the findings, we propose to actively engage with families to identify ways to resolve the tension between their professional roles and gendered family roles. We also propose to explore public-private-partnerships in primary healthcare, so that some of the disparities in government and private salaries could be resolved, and optimize primary care nurses' availability in rural areas.

### Implications on Policy and Practice

In India, rural-urban divide in access to healthcare and health outcomes is related to inequitable distribution of skilled and motivated human resources. The experience and evidence presented here suggests that the rural pathway approach, centered on task redistribution can help in bridging this divide. While recent guidelines in India [as part of Clause 32 of the National Medical Commission Bill (29)] have envisaged the role of nurses (as mid-level providers) in provision of comprehensive primary care in rural areas, it does not provide the pathways to enable them to do so. Our analysis provides some pointers. Sourcing nurses from rural and tribal communities (rather than from urban areas) to serve in rural areas is likely to be more effective since they are more likely to stay and be more empathetic to the communities they serve. A task redistribution approach, where tasks are redistributed between nurses and physicians, who work as a team with a shared responsibility, is likely to be more effective than a task shifting approach, which merely shifts the responsibility to deliver primary health care to nurses alone.

While the government has emphasized on higher remuneration to increase the nursing workforce, the current structure of hierarchy within medical practice does not foster a team approach. An emphasis on building non-hierarchical primary healthcare teams that draws on complementary strengths of PHC nurses and physicians will go a long way in advancing rural healthcare. Several forms of support systems, such as close mentoring, ensuring basic amenities, and good living and working conditions would be required to enable PHC nurses to play an effective role in delivering rural healthcare.

The rural pathway framework ordinarily focuses on systemic processes that can be influenced to strengthen the workforce. It does not encompass the structural factors, such as gender roles and family and societal expectations from the providers, which appear to significantly affect their availability, retention, and performance in rural areas. Future scoping and research should consider these factors while expanding existing pathways for rural workforce.

With dearth of studies in LMICs, the paper provides insights and evidence on a rural pathway approach to enhancing the role of PHC nurses in delivering primary healthcare in rural areas. Such a framework can be used in different contexts to plan and evaluate human resource interventions for improving rural healthcare.

### Limitations of the Study

The study is based on experience of one organization in a rural, tribal area and therefore may not be generalizable to all such areas. It does however provide a framework for understanding the factors that would enable nurses in other similar areas to effectively provide primary healthcare.

We accepted the reasons that nurses stated for leaving the job, but did not conduct interviews with them to understand in-depth nuances of each case. However, the factors that emerged are helpful to design program level interventions to address retention.

Finally, the study did not measure quality of care provided by the nurses. While the large numbers of patient-visits managed by the nurses provide an indication of their acceptance and perceived quality by the patients.

## Data Availability Statement

The raw data supporting the conclusions of this article will be made available by the authors, without undue reservation.

## Ethics Statement

Ethical approval for this study and written informed consent from the participants of the study were not required in accordance with local legislation and national guidelines.

## Author Contributions

AA has contributed to the data analysis and writing of manuscript. MD has contributed to the literature review and writing of the manuscript. SB and PM have reviewed and designed the pathway framework as well as edited the manuscript. All authors have contributed to the conceptualization of the paper.

## Conflict of Interest

The authors declare that the research was conducted in the absence of any commercial or financial relationships that could be construed as a potential conflict of interest.
